# On the Effects of a Rheumatic Vice on the Fibrous and Osseous System

**Published:** 1817-06

**Authors:** Baron Larrey


*For the London Medical and Physical Journal.*

[It has lately been much the custom to speak of Rheumatism in the Heart. To us, it has always appeared, that the more correct language would have been, Translation of Inflammation from the external Muscles to those of the Heart. The following paper shows, that this translation or sympathy is not always to a muscular part.—Ed.]

### *On the Effects of a Rheumatic Vice on the Fibrous and Osseous System.* By Baron Larrey.

IF it be very difficult to explain the action of the causes of internal aneurisms, and those of the cessation of hæmorrhages, it is still more difficult to account for the disagreeable effects produced by rheumatism on the fibrous and osseous system, especially in subjects in whom nature had not terminated the process of ossification. Notwithstanding the obscurity of the steps of this disorder, and the variety of the symptoms, I am of opinion, that the rheumatic principle deprives, by its deleterious properties, the fibrocartilaginous substance, and the osseous vessels, of their vitality. This substance loses its powers and its organic sensibility; sibility; the lymphatic vessels, (which are not accessible to the action of the morbid cause, from which they only appear to receive a sympathetic irritation,) absorb with a new force the molecules of these substances, become (if we may be allowed the expression) inert, and wear away or devour them by degrees; they thus operate a deperdition of substance in the solid bodies, and on the points surrounding the seat of the disorder; and a latent inflammation, accompanied with a kind of serous or purulent secretion which produces deposits by congestion. The residence of this heterogeneous fluid existing in the deteriorated part of the bone, where the ligamentary cartilages favor the progress of the caries, and extend the disorder profoundly; the progress of this internal caries and decomposition is the more rapid where the ossification has not yet been completed by nature. The absorbent system in young subjects acts with great energy, and the phosphate of lime readily abandons the bones, because they have not yet acquired the density necessary to resist the direct action of the virus, or the indirect one of the lymphatic vessels. The patients at the hospitals that we have attended in our practice, were all young men from eighteen to twenty. In some, the rheumatic affection determined improperly the caries of the osseous pieces of the orbicular articulations, or *ginglymoides*, rarely with a spontaneous luxation. In others, the caries of one or two of the vertebræ; and, in a few, the separation of the bones of the pelvis. We shall give examples of each.

This rheumatic virus fixes itself in such or such a part, according to a predisposition in these parts, or to concomitant causes which fix the morbid principle in this point. In every case, the progress of the disorder presents analogous phenomena, with some differences according to its seat, which Pott and the Memoirs of the Royal Academy of Surgery have amply detailed; in general, the disorder is mortal, when it is not properly attended to in time. I have obtained considerable success in applying remedies, but little used, with vigor and perseverance; and, I am persuaded, that if administered on the appearance of the first symptoms, the cure is easy and certain.

The mode of treatment I employ, is to excite the action of the parts affected so as to re-establish that of the weakened vessels, and to restore their proper and natural sensibility.

Cupping, if there be the least sign of local turgescence, Moxa, and the actual cautery, as a topic, have produced, in cases considered hopeless, the happiest effects. Mercurial frictions made as near as possible to the part, and at four or

 five five days' intervals, have appeared to me greatly to contribute to the cure of the patient. I will suppose even that there does not exist any syphilitic vice. I have remarked, in general, that this remedy produces excellent effects in all the disorders improperly called lymphatic, if the patients be not already exhausted.

When the abscess has manifested itself on one of the superficial points, and corresponding with the purulent matter in the seat of the disorder, it may be attacked with success; when, after the use of internal and external remedies, this deposit rests in the same state, it is a sign that the progress of the caries is suspended. In this supposition, I plunge in its whole depth a pointed sharp steel instrument, heated to a white heat, and extract as much matter as possible by dry cupping, which embraces the whole tumor and the two orifices, I then pass a seton of the threads of old linen through the two orifices, and on it a compress steeped in camphorated oil of camomile, and a compressive bandage.

Contrary to the opinion of many surgeons, I endeavour to evacuate all the liquid in the first moments of the operation, and without bearing the effects of the air, which would be more injurious if the pus, retained in any quantity in the purulent space, received its impression, because it would rapidly decompose it, and infect the individual; while, on taking the precaution to evacuate it immediately, we prevent this inconvenience and danger. The application of moxa on the points corresponding with the caries, together with local mercurial frictions, and, internally, sudorifics combined with bitters, sometimes with bark. Sóme mercurial salts, which are administered with more advantage separately and in proper vehicles, are the remedies which I have found to succeed the best. I shall not insist, at length, on the properties of these medicaments, which have been already satisfactorily treated of by Dr. Salmade;* but, for my part, I regard topicals, or the means furnished by surgery, as the most efficacious in this disorder wherever it is susceptible of cure.

We may indulge this hope: 1st, when it is not in a very advanced state, and within the reach of the aid of art. 2d, When the patient is young and exempt from any other virus, as venereal or scrofulous. 3d, When the caries is not profound, and is distant from the internal organs.

It may be easily conceived, that, on the first appearance

* Precis d'observations pratiques sur les maladies de la lymphe ou affections scrofuleuses et rachetiques.

of of the symptoms, the disorder may be attacked with success; but, how shall we explain the success obtained, when a deposit is formed, and probably the caries has began? It is a problem difficult, and perhaps impossible, to solve. I shall, therefore, content myself with narrating faithfully such observations as have come under my notice.

One of the first examples was a young soldier of the Imperial Guard, who entered the hospital after our return from the campaign of Austerlitz, with all the symptoms of a spontaneous luxation, commencing in the left femur at the coxo-femoral articulation; such as acute local pain, want of motion in the limb, its length unnatural,* the trochanter salient, the pining of the extremity, and feverish affection. After some preparations, I administered the tonics and depuratives already mentioned in beverages and draughts, and I hastened to apply all round the articulation, cupping, then light blisters, and a sufficient number of moxas, the ulceration from the burns of which, I always prevent by the immediate application of ammonia.

The symptoms calmed by degrees, and at the ninth application of burning moxa had entirely disappeared; yet I continued the moxa, in order to be more certain of the cure, as well as mercurial frictions round the parts, which I have found to be highly useful.

Eleven subjects of the same age entered the same hospital in succession, afflicted with the same disorder, which had developed itself at the articulation of the thigh or the knee: five of them were nearly cured, they limped a little, and the limbs were shortened from one-third to two-thirds of an inch. In three others, the spontaneous luxation appeared to be established; that is to say, the signs indicated by authors to characterize this luxation existed; such as a sensible shortening of the member, the deviation of the foot or knee generally outwards, the elevation of the great trochanter, stiffness and difficulty in the motion of the thigh, and particularly the impossibility of bending it.

Is it the head of the femur intact which, really abandoning the cotyloid cavity, fixes itself on the edge of this cavity, equally intact? or, is it a waste or reduction of the head of this bone? or, in fine, an excavation more or less deep in the articular cavity, which produces the shortening and the deformity of the limb?—My observations lead me to

* The translator witnessed a similar case now treating by Baron Larrey at the Hospital of the Royal Guard, the length of the leg was increased two inches.

adopt adopt the latter opinion, as that which agrees the best with the principles of sound physiology. Besides, I have never been able to verify, after the death of the persons who were supposed to have suffered this spontaneous luxation, the pretended displacing of the head of the femur, that authors announce by the choaking up, or successive thickening, of the cartilages of the cotyloid cavity, which expels by degrees the head of the humerus, till, at length, the luxation is complete. If these circumstances be ever met with, we may affirm, that the luxation has been accidental, that is to say, produced by a determinate external cause, which may be unknown, perhaps, even by the patient.

The diarthrosial cartilages are unsusceptible of any of the alterations proper to the organized parts, and, far from acquiring a greater thickness or larger size, they easily dissolve into little friable grains, more or less pulverulent, and are easily absorbed by the lymphatics, so as to entirely disappear, as I have regularly observed on opening the bodies of the patients who have died under this disorder in the articulation. This cartilaginous substance being no longer secreted by the osseous vessels of the extremities of the bones it meets with, its particles are disseminated, become decomposed, and disappear by the effect of absorption, which already reduces the length of the femur, and thereby shortened the member. The osseous vessels even flatten, acquire density, and become reduced, which tends still more to shorten the member; and all this may and does take place without any serious concomitants, as inflammation, suppuration, abscess, or hectic fever; we find, in fact, very frequently osseous articular pieces which had been attacked by the disorder long before, and entirely deprived of cartilages without their having been deprived of the motion proper to these articulations. I possess several pieces of this nature, and many more are to be found in anatomical cabinets; but a very interesting case, which I will relate, proves, that the cartilaginous substance may disappear, and be reestablished, without producing any of those serious results which generally happen in disorders of the joints, when abandoned solely to the resources of nature.

In the anatomical cabinet of Vienna is a dissected thorax, in which was shown me the head of the right humerus, confined between the second and third of the true ribs, the whole of the orbicular mass being in the cavity of the breast. This singular transposition of the osseous extremity was the result of an accidental luxation, by the person, æt. 17, falling from his height on his elbow, the arm extended in a direction direction from the body; the head of the humerus, after having torn the capsula, was driven with violence in the hollow of the arm-pit under the pectoralis, in such a manner as to separate the two ribs and pass through the interval; the diameter of the sphere of the head of the humerus, having overcome the obstacle, penetrated entirely into the thoracic cavity, driving before it the corresponding portion of the pleura. Every effect to reduce this extraordinary luxation was in vain, dangerous symptoms manifested themselves, which bleeding, baths, and restoratives, at length overcame; but the arm remained separated from the body in the same state, to which the patient accustomed himself by degrees; After a few years of suffering, he was no longer incommoded by it, and lived with this infirmity to the age of thirty-one, when a complaint of a different nature carried him off. The physicians, wishing to know the precise nature of this infirmity, which they could only judge of imperfectly, opened the body, and were much surprised to find the head of the humerus in the breast, enveloped by the pleura, and firmly pressed by the two ribs, between which it had entered; they were still more astonished, when, instead of finding a hard spherical body covered with a cartilage, they discovered only an exceedingly soft membranous body, yielding to the slightest pressure of the finger.

The cartilage and the osseous tissue of all that portion of the humerus contained in the cavity of the breast, had entirely disappeared, the absorbents had decomposed it, and, like faithful guardians, had sought to destroy by degrees, as they would not expel in a mass, an enemy whose presence incommoded and was injurious to the regular operation of their functions. There remained nothing of the head of the humerus but these membranous rudiments, which even belonged in a great measure, in my opinion, to the pleura costalis. This example, in supporting my opinion of the nature of the morbid action in rheumatic disorders of the joints, shows the powers of nature in decomposition, which I am inclined to believe equally rich in those of regeneration and recomposition.

*Paris; April* 1817.

(To be continued.)

